# Complete mitochondrial genome of the longhorn date palm stem borer *Jebusaea hammerschmidtii* (Reiche, 1878)

**DOI:** 10.1080/23802359.2021.1989334

**Published:** 2021-10-15

**Authors:** Guilherme B. Dias, Ahmad M. Aldossary, Hamadttu A.F. El-Shafie, Fahad M. Alhoshani, Mohamed B. Al-Fageeh, Casey M. Bergman, Manee M. Manee

**Affiliations:** a Department of Genetics and Institute of Bioinformatics, University of Georgia, Athens, GA 30602, USA; b National Center for Biotechnology, King Abdulaziz City for Science and Technology, Riyadh, Saudi Arabia; c Date Palm Research Center of Excellence, King Faisal University, Al-Ahsa, Saudi Arabia; d Life Sciences and Environment Research Institute, King Abdulaziz City for Science and Technology, Riyadh, Saudi Arabia; e National Center for Bioinformatics, King Abdulaziz City for Science and Technology, Riyadh, Saudi Arabia

**Keywords:** mtDNA, date palm stem borer, longhorn beetle, Coleoptera, Cerambycidae

## Abstract

The 15,619 bp mitochondrial genome of *Jebusaea hammerschmidtii* was assembled from short reads, annotated, and compared to the genomes of other longhorn beetles (Cerambycidae). Gene content was typical of animal mitochondrial genomes and contained 13 protein-coding, 22 tRNA, and 2 rRNA genes. Gene organization was identical to that of other longhorn beetles. Phylogenetic analysis placed *J. hammerschmidtii* within the subfamily Cerambycinae, and strongly supported the monophyly of the Cerambycinae, Lamiinae, and Prioninae subfamilies.

*Jebusaea hammerschmidtii* (Reiche, 1878) (Coleoptera: Cerambycidae) is an important pest of the date palm (*Phoenix dactylifera*) in the Middle East and North Africa (El-Shafie et al. 2017). Females lay eggs at the base of palm fronds or in cracks on the tree trunk. After hatching, larvae bore into the tree, causing physical damage as they develop (El-Shafie 2015). As of May 2021, a single nucleotide record was available in GenBank for *J. hammerschmidtii* (COI, MG564344.1). Here we sequenced, assembled, and annotated the complete *J. hammerschmidtii* mitogenome and compared it to the mtDNAs of other longhorn beetles.

A single unsexed larva was obtained from a naturally infested date palm in the orchard of the Date Palm Research Center of Excellence at King Faisal University, Al-Ahsa, Saudi Arabia (25°16′04.8″N 49°42′25.2″E). The larva was identified as *J. hammerschmidtii* by specialists on the basis of its morphology and the fact that no other species with similar morphology occurs in this region. The entire specimen was utilized for DNA extraction and thus not deposited in a collection. DNA purification followed the ‘salting-out’ protocol (https://support.10xgenomics.com/permalink/7HBJeZucc80CwkMAmA4oQ2). DNA was cleaned using AMPure XP beads and 0.6 ng was utilized for barcoding and library construction using the Chromium Genome Reagent Kit Protocol v2 (RevB). The library was sequenced on a Illumina NextSeq 500 mid-output flow cell with 150 bp paired-end reads. Resulting fastq files were processed with LongRanger v2.2.2 (basic pipeline) (Zheng et al. 2016) to remove barcodes, then de-interleaved using ‘reformat.sh’ from BBMap v38.83 (Bushnell 2014). The mitogenome of another Cerambycinae beetle, *Xylotrechus grayii* (KM112084), was downloaded using ncbi-acc-download v0.2.5 (https://github.com/kblin/ncbi-acc-download) and used as mapping seed to identify mitochondrial sequences in the *J. hammerschmidtii* short-read dataset. We used two passes of GetOrganelle v1.7.3.2 (Jin et al. 2020a) to assemble the *J. hammerschmidtii* mitogenome (1st pass, ‘-w 111 -R 10 -F animal_mt’; 2nd pass, ‘-w 100 -R 15 -F animal_mt’). The first pass was used to identify all read pairs that matched the *X. grayii* mtDNA or the animal mtDNA database used by GetOrganelle. In the second round, mtDNA-matching read pairs were used as input and the entire mtDNA was recovered as a circular molecule of 15,619 bp. *J. hammerschmidtii* mtDNA was annotated with GeSeq v2.03 (Tillich et al. 2017) with available Cerambycinae mtDNAs as annotation references (*Aeolesthes oenochrous, Massicus raddei, Neoplocaederus obesus, Epipedocera atra, Nortia carinicollis, X. grayii, Xystrocera globosa*). ARWEN v1.2.3 (Laslett and Canbäck 2008) and tRNAscan-SE v.2.0.7 (Chan and Lowe 2019) were used for tRNA prediction with default settings. Redundant tRNA predictions were removed and the protein coding gene annotation was curated manually. Ribosomal RNA genes were extended after annotation with MITOS web server (commit 6b33f95) (Bernt et al. 2013). The final annotation revealed a gene content and organization identical to other Cerambycidae beetles, with 13 protein coding genes (PCGs), 2 ribosomal RNAs, and 22 transfer RNAs (Accession MZ054170).

Out of 12 PCGs for which start codons were identified, five used ATT (ATP8, ND2, ND3, ND4L, and ND5), three used ATG (COX3, CYTB, and ND4), two used ATC (COX2 and ND6), one used ATA (ATP6), and one used TTG (ND1). The start codon for COX1 could not be determined. The TAA stop codon was identified for 4 PCGs (ND4L, ND6, ATP6, and ATP8), and an additional 6 PCGs had an incomplete stop codon (T) hypothesized to form TAA by polyadenylation (ND2, ND4, ND5, COX1, COX2, and COX3). Three PCGs had a TAG stop codon (ND1, ND3, and CYTB). All tRNAs were predicted to form cloverleaf secondary structures.

A total of 24 Cerambycidae mtDNA genomes plus an outgroup from the Chrysomelidae subfamily (*Chrysomela vigintipunctata*) were retrieved from RefSeq and used to place our *J. hammerschmidtii* mtDNA genome in a phylogenetic context (Kim et al. 2009; Chiu et al. 2016; Li et al. 2016; Wang et al. 2016; Jin et al. 2017; Liu et al. 2018; Que et al. 2019; Wang et al. 2019a, 2019b, 2019c, Dai et al. 2020; Li and Lu 2020; Lin et al. 2020; Su and Wang 2020; Yan et al. 2020; Jin et al. 2020b; Dong et al. 2021a, 2021b). Protein sequences from each mitogenome were concatenated and aligned using MAFFT v.7.455 (Katoh and Standley 2013), and a maximum likelihood phylogeny constructed using IQ-TREE v.2.1.2 (Minh et al. 2020) with the *mtZOA*+F + R5 model using *C. vigintipunctata* as the outgroup for rooting the tree. Node support was calculated after 10,000 ultrafast boostrap replicates. *J. hammerschmidtii* clusters with *X. grayii* with a bootstrap support of 68 (Figure 1). Despite the low support for the grouping of *J. hammerschmidtii* and *X. grayii*, the clade containing *J. hammerschmidtii*, *X. grayii*, *N. carinicollis*, and *E. atra* has bootstrap support of 99. Monophyly of subfamily Cerambycinae, which contains *J. hammerschmidtii*, is recovered with bootstrap support of 98. The phylogeny supports monophyly of Prioninae and Lamiinae subfamilies with bootstraps of 100 in both cases, corroborating previous studies (Nie et al. 2021) (Figure 1). Future analyses with denser taxon sampling will help elucidate the tribe-level phylogeny of longhorn beetles.

**Figure 1.**
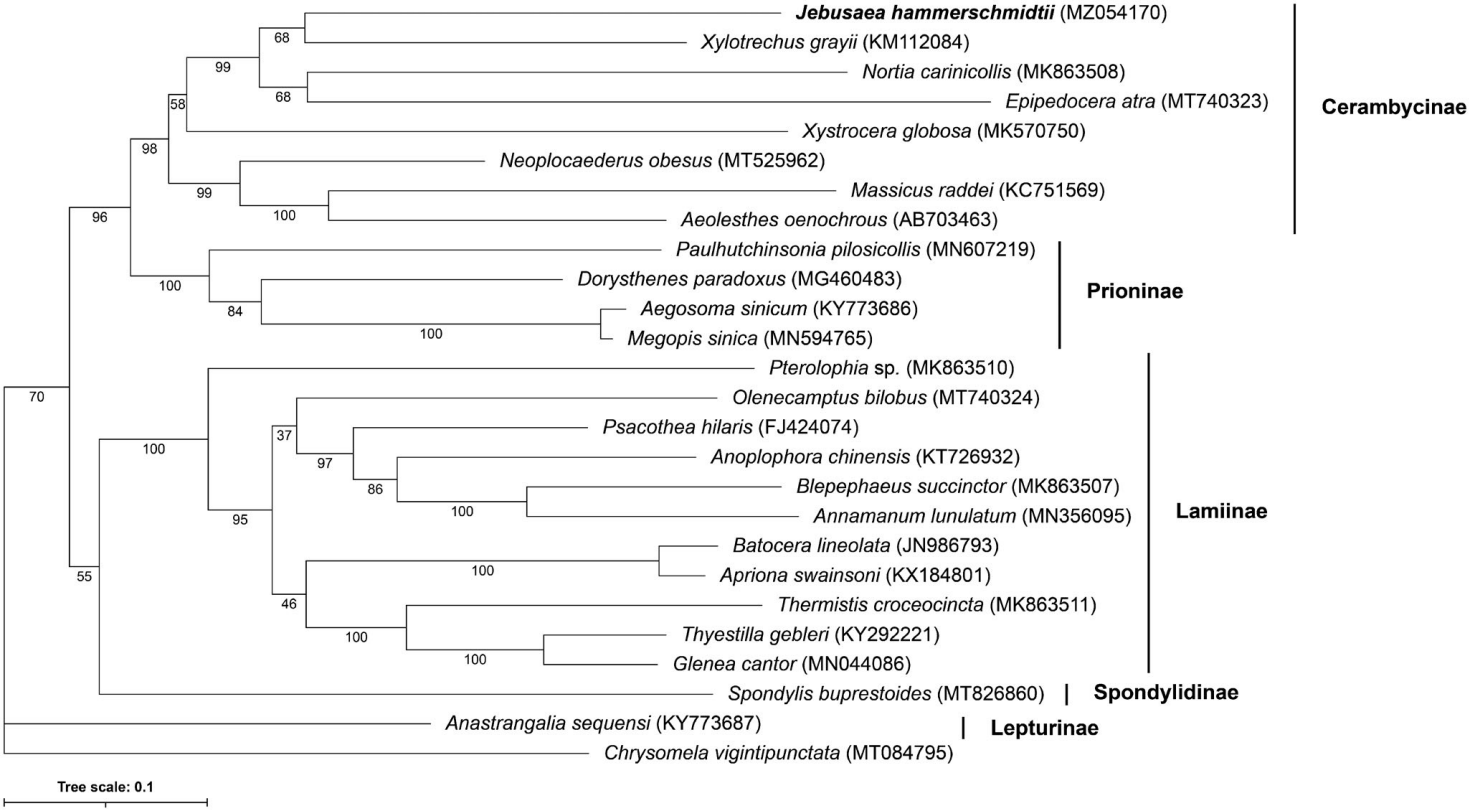
Maximum Likelihood phylogenetic tree of Cerambycidae beetle mtDNA genomes. Accession numbers are given after each species name. Numbers below each node represent ultrafast bootstrap support values after 10,000 replicates. The tree was rooted by setting *Chrysomela vigintipunctata* as the outgroup taxa.

## Data Availability

The annotated mitochondrial genome described here is available in GenBank under accession MZ054170 (https://www.ncbi.nlm.nih.gov/nuccore/MZ054170). Raw mitochondrial reads used for assembly are available in the SRA under accession SRR14321410 (https://www.ncbi.nlm.nih.gov/sra/SRR14321410).
